# Night and evening shifts and risk of calling in sick within the next two days – a case-crossover study design based on day-to-day payroll data

**DOI:** 10.5271/sjweh.4074

**Published:** 2023-02-27

**Authors:** Ann Dyreborg Larsen, Helena Breth Nielsen, Jonas Kirschheiner-Rasmussen, Johnni Hansen, Åse Marie Hansen, Henrik Albert Kolstad, Reiner Rugulies, Anne Helene Garde

**Affiliations:** 1 The National Research Centre for the Working Environment, Copenhagen, Denmark; 2 Danish Cancer Society Research Center, Copenhagen, Denmark; 3 Department of Public Health, University of Copenhagen, Copenhagen, Denmark; 4 Department of Occupational Medicine, Danish Ramazzini Centre, University of Aarhus, Aarhus, Denmark

**Keywords:** hospital staff, irregular work hour, night shift, register data, sickness absence, shift work, shift worker

## Abstract

**Objective:**

Night and evening work is associated with risk of sickness absence, but little is known about the acute effects of these types of shifts on sickness absence. The aim of the current study is therefore to examine the risk of calling in sick within two days after a night or an evening shift.

**Methods:**

By use of a case-crossover design, odds of calling in sick within two days after a night or an evening shift compared to day shifts were analyzed within the same person. Day-to-day information on shifts and sickness absence were derived from the Danish Working Hour Database on 44 767 cases. Data were analyzed using conditional logistic regression. The analyses were supplemented by extensive testing of methodological choices.

**Results:**

Analyses showed higher odds of calling in sick after a night shift [odds ratio (OR) 1.22, 95% confidence interval (CI) 1.14–1.30] and lower odds after an evening shift (OR 0.89, 95% CI 0.84–0.93) compared to day shifts within the same person. Testing of methodological choices suggested that in particular the duration of case and control periods, time between these periods along with the number of control periods affected the results.

**Conclusion:**

This large and unique within-person study among Danish hospital employees indicate that the risk of calling in sick is affected by the types of shifts, independently of sex, age, and time-invariant confounding. Extensive testing identified important methodological choices eg, length and number of included periods to consider when choosing the case-crossover design.

A significant proportion of the workers in the EU work nights (19%) and/or in shifts (22%) (1). In the Danish hospital sector, it is estimated that approximately 24% of workers have schedules including evening shifts without nights, and 11% work night shifts permanently or as part of 3-shift schedules (10.2%) (2). Previous studies have suggested that night and shift work are associated with an increased risk of certain cancers, ischemic heart disease, and diabetes (3–5).

Sickness absence is a predictor of subsequent morbidity, dissatisfaction, disability, and mortality (6–8) and is often used as an indicator for work-related health (9). In addition, sickness absence poses a daily organizational challenge to many workplaces, where substitutes are needed to cover the staffing requirements.

A systematic review by Merkus et al (10) concluded in 2012 that epidemiological evidence was inconclusive regarding the impact of rotating shifts, night work, and fixed night work on the risk of sickness absence. Further, the authors called for more detailed and non-self-reported exposure data. Since then, several studies have included more detailed and register-based exposure data, eg, payroll data when studying the association between night or evening work and sickness absence (11–19). Yet, the results of these studies are inconsistent. Some studies found higher rates of sickness absence among night shift workers (11–16, 18) compared to workers on day shift, whereas other studies observed no relationship (12, 17, 19).

There are several explanations for the inconsistencies: the studies used different lengths of sickness absence eg, short-term (1–3 days) (15, 16, 18), 1–8 days (12) or long-term (>3–30 consecutive days) (11–14, 19). This makes it difficult to compare results as there may be different mechanisms related to short- and long-term sickness absence (20). Further, specifying the length of sickness absence may compromise basic epidemiological principles in regards to conditioning on a descendant of the outcome (21). In addition, other methodological challenges such as the high frequency of the outcome make sickness absence a challenging outcome to study with the traditional survival analysis with long follow-up as most of the study population will experience sickness absence at some point in time during observation. The risk of sickness absence is also highly correlated to previous sickness absence (22) and inversely correlated to working time, as one cannot register sickness absence and be working at the same time. Sickness absence is associated with many other factors, which can be difficult to obtain information on when using register data eg, personality, genetic background and to some extent work environment (23–26). Studies on sickness absence are therefore vulnerable to between-subject differences ie, that the risk of sickness absence is not the same in the exposed group as in the reference group. These challenges can be addressed by use of a case-crossover design, which handles differences between employees by self-matching and thereby excludes effects of time-invariant covariates (27). This design has been used in Finnish studies (15, 16, 18) to study the effects of work schedules within the past 28 days on sickness absence, but no studies have yet investigated acute effects of night or evening shifts and the risk of sickness absence.

Against this background, we aim to examine if night or evening shifts are associated with calling in sick within the next two days, using a case-crossover design in a large Danish cohort based on day-to-day pay-roll data.

## Methods

### Study design

We applied a case-crossover design with a unidirectional approach and multiple control intervals. We analyzed data from the Danish Working Hour Database (DWHD) a Danish nationwide database including administrative payroll data from 2007 onwards. The database holds detailed information on actual working hours from >340 000 employees from the five Danish regions, including all public hospitals. For all employees, we had precise information on daily starting and ending time for each shift worked as well as age and sex along with variables related to the specific employment conditions of the employee. In addition, the database also includes day-to-day information on sickness absence. Further information on the DWHD is published elsewhere (2).

### Case selection

**Figure 1:**
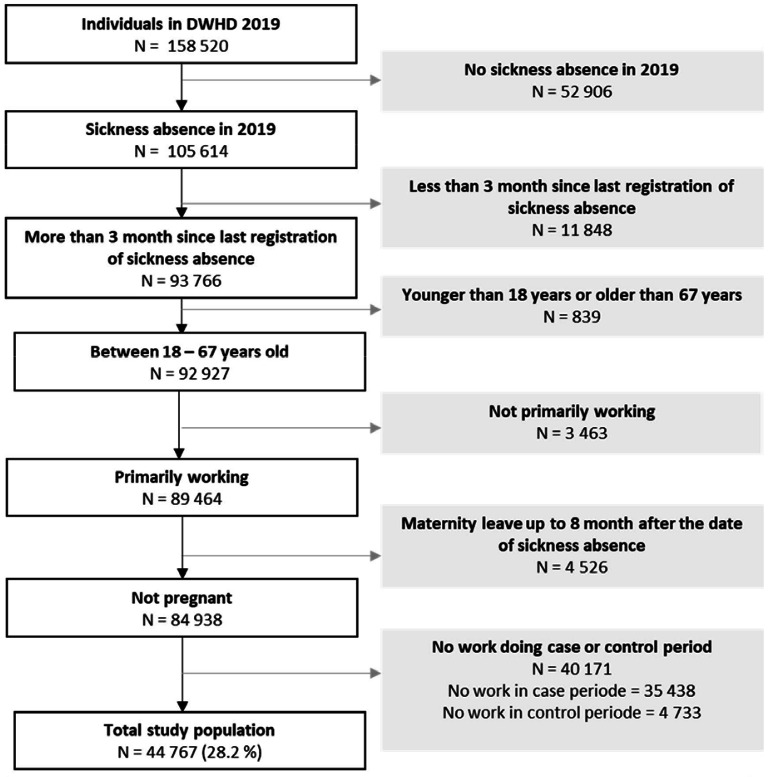
Flowchart of the study population.

We restricted the study population to adults below the general retirement age (18–67 years old) in 2019 with ≥50% employment time were included. This allowed us to include participants with part-time jobs, which is quite common among hospital workers in Denmark, but exclude participants with low employment degrees (marginal part-time work) eg, due to health issues, which evidently could affect their sickness absence. We also excluded those who were pregnant by excluding women on parental leave within the 8 months after the sickness absence as risk of sickness absence differs across the pregnancy (28). Only employees with a change in exposure (day, evening or night) between the case and the control period(s) were included. We identified 44 767 cases according to the definition (see figure 1).

### Exposure assessment – night and evening work

We defined night work as ≥3 hours of work between 23:00 and 06:00 similar to previous studies (29). Evening shifts were defined as 3 hours of work between 18:00 and 02:00. Day shifts were defined as shifts starting after 06:00 and ending before 21:00. The definitions of shifts were not mutually exclusive: night work was given the highest priority, then evening work and the lowest priority was day work. The exposure assigned to each case and control period were the most recent shift to end or start within the case or control period. If the employee was sick listed during work hours in the case period, the most recent shift before was considered as the exposure in the period.

### Cases – sickness absence

**Figure 2:**
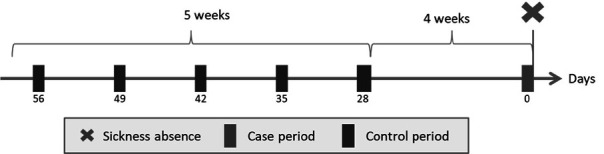
Illustration of the case-crossover study design. The case period (with the circle) covers 48 hours preceding a sick leave registration (the X). Five control periods of 48 hours (prior square boxes) were matched by weekday 28–56 days prior to the sick leave registration.

Information on daily sickness absence was drawn from DWHD. The cases were selected as each person’s first day of sick-leave registration preceded with ≥90 days without sickness absence after 1 January 2019. The case period was defined as the two days leading up to the day of the sick-leave registration. Control periods were matched to the case period by weekday, to adjust for differences across days of the week. Thus, for each case period we selected all (up to five) two-day control periods occurring day 28–56 before the case period. We included only control periods that were followed by a day with work, respectively 28, 35, 42, 49 and 56 days before the day of sickness absence (see figure 2), as sickness on days off are not registered in our payroll data.

### Covariates

Sex (woman/man) and age groups (18–24, 25–34, 35– 44, 45–54, ≥55 years old) from DWHD were included as effect modification and used in stratifications.

### Statistical analyses

The case-crossover design is a type of fixed effects models (30). Only employees with a change in exposure between the case and control period (discordant pair) contribute to the analysis (31, 32). Discordant *exposed pairs* were calculated as the total number of pairs where employees were exposed in the case period and unexposed during the control period. Discordant *unexposed pairs* were calculated as the number of pairs where the case period was unexposed and the control period was exposed. Also, the number of employees with at least one discordant pair was calculated.

The case-crossover matched-pair interval approach was used to compare each employee’s exposures in the case period with exposures in the control periods. Using conditional logistic regression with the employee used as strata, we calculated odds ratios (OR) with 95% confidence intervals (CI). All analyses were conducted in SAS 9.4 (SAS Institute, Cary NC, USA) with Proc Logistic in accordance with previously described techniques (33).

*Main analyses*. We compared the exposure in the reference periods (control periods) with the exposure before calling in sick (case period). Exposure was measured as having a night or an evening shift within two days of the reference. Day shifts in the case period were used as reference.

The analyses were carried out in the total study population and stratified populations in regards to sex and age groups. Further we compared risk of calling in sick after a second, third or fourth consecutive night shift (exposure) to the first night shift (reference).

*Sensitivity analyses for check of methodological choices*. All methodological choices were made *a priori*. Therefore, to gain insight into the consequences of the epidemiological and methodological decisions of the design, we performed sensitivity analyses of night shift to test: (i) effect of the duration of the case and control periods, the main analysis (night versus day) was repeated with a case and control periods of 24 hours and 72 hours; (ii) the effect of number of control periods, the main analysis was repeated with 1, 3, 6 and 9 control periods; (iii) the effect of days between control periods, the main analysis was repeated with 5-day intervals and 5 days between case and control in one analysis, and with 10 days intervals and 14 days between case and control periods in another analysis; (iv) the effect of excluding participants with sickness absence 90 days before case periods, the main analysis was repeated while including all participants regardless of preceding sickness absence, control periods with sickness absence were treated as cases; (v) if degree of full-time/part-time work affected the results, the main analysis was repeated with >75% and 100% employment, respectively.

As previous studies have been conducted on nursing personnel only, looking into short-term sickness absence (1–3 days) (15, 16, 18) the main analyses were repeated restricted to nursing personnel and sickness absence up to three days.

To test robustness of the main analyses, we repeated the setup from the main analyses but used non-night shift (day and evening combined) as reference.

## Results

The studied population included primarily women (79.7%) (table 1) with few subjects <24 years (2.7%). In the remaining of the age groups, the distribution was similar (22.0–26.4%). Most participants worked as nursing personnel including nurses, nurse assistants, midwives etc (51.7%), but also as physicians (13.1%) and other professions including physical therapists, occupational therapists etc (35.2%).

**Table 1:** Characteristics of employees in the main analysis.

	Cases (N=44 767)	

	N	%
Age (years)		
18–24	1194	2.7
25–34	9840	22.0
35–44	11 440	25.6
45–54	11 832	26.4
55–67	10 461	23.4
Sex		
Women	35 658	79.7
Men	9109	20.4
Occupation		
Physician	5843	13.1
Nurse	23 124	51.7
Others	13 501	30.7
Missing	2299	5.1

### Results from the main and supplementary analyses

Table 2 shows the assessment of risk of sickness absence after a night or evening shift (the exposures) compared to a day shifts (the references). Results from the analyses on all employees showed higher odds of sickness absence after a night shift compared to a day shift (OR 1.22, 95% CI 1.14–1.30) Evening shifts were associated with lower odds of sickness absence (OR 0.89, 95% CI 0.84–0.93) compared to day shifts.

**Table 2:** Case-crossover design ^a^. Conditional logistic regression analyses of risk of sickness absence after a night or evening shift (exposure) compared to the risk after a day or non-night shift (reference). [D=discordant; OR=odds ratio; CI=confidence interval]

Work shift characteristics	Exposed in case period	Unexposed in case period	D-exposed pairs ^b^	D-unexposed pairs ^c^	D-cases ^d^	Conditional logistic regression analysis		

						OR	95% CI	P-value
All employees								
Night vs. day (ref)	3352	37 003	3294	2702	1844	1.22	1.14–1.30	<0.001
Evening vs. day (ref)	4412	37 003	4847	5433	2720	0.89	0.84–0.93	<0.001
2^nd^ vs. 1^st^ night shift	1765	4024	563	514	418	1.13	0.99–1.30	0.070
3^rd^ vs. 1^st^ night shift	1306	4024	346	237	208	1.44	1.20–1.73	0.001
4^th^ vs. 1^st^ night shift	444	4024	150	111	97	1.31	0.99–1.73	0.06
Sex								
Men								
Night vs. day (ref)	769	7524	841	604	421	1.38	1.20–1.58	<0.001
Evening vs. day (ref)	816	7524	910	1052	395	0.86	0.77–0.97	0.020
Women								
Night vs. day (ref)	2583	29 479	2453	2098	1423	1.18	1.09–1.27	<0.001
Evening vs. day (ref)	1596	29 479	3937	4381	2225	0.89	0.84–0.94	<0.001
Age (years)								
18–24								
Night vs. day (ref)	115	909	134	81	76	1.60	1.14–2.27	0.009
Evening vs. day (ref)	170	909	206	231	118	0.90	0.70–1.15	0.400
25–34								
Night vs. day (ref)	1090	7653	1287	1056	727	1.22	1.10–1.35	<0.001
Evening vs. day (ref)	1097	7653	1408	1526	774	0.91	0.83–1.01	0.07
35-44								
Night vs. day (ref)	834	9720	905	682	487	1.27	1.12–1.44	<0.001
Evening vs. day (ref)	886	9720	1033	1078	572	0.95	0.85–1.07	0.38
45–54								
Night vs. day (ref)	748	10 018	638	556	362	1.19	1.02–1.38	0.02
Evening vs. day (ref)	1066	10 018	1150	1384	642	0.82	0.74–0.91	<0.001
55–67								
Night vs. day (ref)	565	8703	330	327	192	1.06	0.87–1.29	0.60
Evening vs. day (ref)	1193	8703	1050	1214	614	0.86	0.77–0.96	0.96

a Case-crossover design with up to 5 control periods per case period.

b Discordant exposed pairs: number of pairs where case is exposed and control is unexposed.

c Discordant unexposed pairs: number of pairs where case is unexposed and control is exposed.

d Discordant cases: number of employees with at least one discordant pair.

Table 2 shows the odds of sickness absence after consecutive night shifts compared to the odds of sickness absence after the first night shifts. Results show that the highest odds was after the third night shift in a row compared to the first night shift (OR 1.44, 95% CI 1.20–1.73).

When stratifying by sex, men’s risk estimates were higher than women’s. However, both sex showed similar results as in the first analyses: higher odds of sickness absence after night shifts compared to day (OR_men_ 1.38, 95% CI 1.20–1.58, OR_women_ 1.18, 95% CI 1.09–1.27) and lower odds of sickness absence after an evening shift (OR_men_ 0.86, 95% CI 0.77–0.97, OR_women_ 0.89 95% CI 0.84–0.94) compared to after a day shift.

When stratifying on age groups the results were similar to previous analyses; higher odds of sickness absence after a night shift in the age groups 18–54 years of age. Further, the analyses showed lower odds of sickness absence after an evening shift compared to a day shift when ≥45 years of age.

### Results from the sensitivity analyses

Table 3 includes all the sensitivity analyses. Results from the main analysis on the total population comparing night to day shifts are included at the top.

**Table 3:** Case-crossover design a. Sensitivity analyses. Conditional logistic regression analyses of risk (OR) of sickness absence with 95% CI. First line presents the results from the main analysis (in italics). [D=discordant; OR=odds ratio; CI=confidence interval]

Analysis	Exposed in case period	Unexposed in case period	D-exposed pairs ^b^	D-unexposed pairs ^c^	D-cases ^d^	Conditional logistic regression analysis		

						OR	95% CI	P-value
Total population	3352	37 003	3294	2702	1844	1.22	1.14–1.30	<0.001
Nursing personnel and short-term sickness absence	2332	16 959	2818	1795	1196	1.08	1.00–1.17	0.06
Duration of periods (days)								
1	2142	31 908	1726	1620	1009	1.06	0.97–1.15	0.22
3	4125	49 635	3722	3239	2289	1.17	1.11–1.24	<0.001
Number of control periods								
1	1765	25 335	664	576	664	1.15	1.10–1.29	0.01
3	2984	31 443	2006	1644	1441	1.21	1.12–1.30	<0.001
6	3446	37 382	3939	3249	1991	1.22	1.14–1.30	<0.001
9	3557	37 876	5832	4817	2273	1.22	1.15–1.30	<0.001
Different lengths between case period and control period (days)								
5	3402	38 036	3174	2971	1966	1.07	1.01–1.34	0.03
10	3407	37 466	2953	2781	1929	1.09	1.02–1.16	0.008
First sickness absence in 2019 as case	3529	39 788	3475	2862	1950	1.21	1.13–1.29	<0.001
Degree of full-time work								
≥0.75	3245	36 028	3270	2674	1826	1.22	1.14–1.31	<0.001
≥1	1770	25 797	2338	1853	1212	1.25	1.15–1.35	<0.001
Night vs non- night (reference)	3352	41 415	3914	3472	2149	1.19	1.12 -1.27	<0.001

^a^Case-crossover design with up to 5 control periods per case period.

b Discordant exposed pairs: number of pairs where case is exposed and control is unexposed.

c Discordant unexposed pairs: number of pairs where case is unexposed and control is exposed.

d Discordant cases: number of employees with at least one discordant pair.

When testing the *duration* of periods, analyses showed that including periods of only one day the OR decreased (1.06, 95% CI 0.97–1.15), whereas the inclusion of three days were close to the main result (OR 1.17, 95% CI 1.11–1.24). When testing the *number* of control periods, results showed that when including only one control period the risk estimate was lower than in the main analysis. However, risk estimates maintained the size regardless of including three, six or nine control periods. There was a slight change in risk estimates when changing the length between case and control periods, where seven days (or the same weekday) showed the highest risk estimates.

It made no difference to the results, if participants with sickness absence 90 days prior to the case period were excluded or not (OR 1.21, 95% CI 1.13–1.29). Similarly the results were not affected when restricting to participants working 75% of full-time (OR 1.22, 95% CI 1.14–1.30) or only full-time (OR 1.25, 95% CI 1.15–1.35).

When restricting to nursing personnel and short-term sickness absence (1–3 days), the results showed the same tendency as the main results but with lower risk estimates. Similarly, when including day and evening shifts as reference (= non-night shifts), the risk estimate was lower, but still showed higher odds of calling in sick after a night shift compared to non-night shifts (OR 1.19, 95% CI 1.12–1.27).

## Discussion

The main analyses showed that the risk of calling in sick was 22% higher after a night compared to day shift within the same person in a population of >44 000 included individuals. The odds of calling in sick were, however, 11% lower after an evening compared to day shift. Further, supplementary analyses showed higher odds of sickness absence after the third night shift in a row compared to the first. When stratifying on sex and age groups, the results from the main analyses were corroborated.

Sensitivity analyses checking the methodological choices showed that changing the duration of the case and control period (1–3 days) and the time between case and control period (5–10 days), had the largest effect on the results in terms of lower risk estimates. In contrast, there was little effect of changes in number of control periods (>5) and degree of full-time employment or when including individuals with previous sickness absence.

### New insight

The study brings new insight to the acute effect of night shift work on risk of calling in sick, as this is the first study to investigate this in a study population beyond pregnant women (28). It is also, to the authors’ knowledge, the first study to observe acute effects of evening shifts on sickness absence. Further, the current study includes a large number of individuals and is the largest study to date to investigate sickness absence due to night shift work using the case-crossover design. The results from the extensive testing of methodological choices indicate that although the case-crossover design meet many of the previous challenges, one should be aware of the consequences of these choices.

### Previous literature

No previous studies have addressed the acute risk of calling in sick after a night shift except a single study on pregnant women which found higher risk of calling in sick after a night shift in the 1^st^ (OR 1.28, 95% CI 1.19–1.37) and 2^nd^ trimester (OR 1.27, 95% CI 1.17–1.39) (28). The results support, to some extent, the findings in the current study. However, as sickness absence during pregnancy may have other reasons or mechanism, these studies are not fully comparable.

An association between night shift work and increased risk of sickness absence in general (not acute) has been observed in other recent studies (11, 13–16, 18). Studies from Finland using the case-crossover design, reported that night shift work during the last 28 days (case exposure window) was associated with increased odds of short-term sickness absence (1–3 days) compared to working time 28 days earlier (case control window) (15, 16, 18). Several of the previous case-crossover studies on night work and sickness absence have been conducted in a study population of nurses only and with short-term sickness absence of 1–3 days as outcome. We therefore repeated the main analysis in a sub-group of nurses limiting sickness absence to 1–3 days. When comparing the results from our main analysis, the OR for the analysis on nurses in this study was lower than for all employees, but still indicating higher odds of calling in sick after a night shift compared to a day shift (OR 1.08, 95% CI 1.00–1.17). The OR for nurses further corroborated previous findings (16) and extended them to another national context. The differences in risk estimates between countries, might be caused by slightly different study designs, and a reflection of contextual differences. In some countries, payment of sickness absence benefits require medical certification – in Finland after three days (16), in Norway after eight days (12) and in Denmark after four weeks (34) – which may affect the incentive for return to work.

We observed lower odds of sickness absence among those working evenings compared to day shifts, which confirms previous findings in a study using the same method (16). It also indicates that night and evening shifts have different mechanisms in regards to sickness absence. Being awake at night and the resulting circadian rhythm disturbances, lack of restitution, and fatigue are the strongest explanatory parameters in relation to night work and the risk of sick leave. However, the same parameters cannot explain the reduced risk of sickness absence when working evening shifts. Rather, we expect evening and day shifts to be comparable in regards to the biological mechanisms and, therefore, in risk of sickness absence. One could speculate that lower demands for amount of work on evening shifts compared to day time work (31) could contribute to reduced sickness absence (and more sickness presenteeism on evening shifts). However we were unable to study this in the current data. In the current study, we included the acute effect of sickness absence, however previous studies have found higher risk of long-term sickness absence when working evening shifts (13, 35).

Ropponen et al (16) found a higher risk of sickness absence after ≥2 consecutive night shifts (OR 1.24, 95% CI 1.12–1.38) and especially after ≥4 consecutive night shifts (OR 1.54, 95% CI 1.10–2.15) indicating a dose–response relationship. This could not be fully corroborated in the current study as results showed only statistically significant results for the third consecutive night shift compared to the first and not for the second or the fourth consecutive shift compared to the first.

Stratification on sex showed that both women and men had statistically higher odds of calling in sick after a night shift compared to day shift. Highest odds were found among men in contrast to a previous case-crossover study (18). The other two case-crossover studies were restricted to women only (15, 16).

Stratification on age groups showed higher odds of sickness absence after a night shift and lower odds after an evening shift compared to day shifts for most age groups. The similar results across age groups is in line with previous case-crossover studies (15, 18).

### Methodological considerations

The use of the case-crossover design enabled us to handle several previous challenges in studies of night shifts and sickness absence. In this design, the participant was compared with herself/himself, minimizing the need for adjustment for time-invariant covariates. With all methodological choices made *a priori*, it is critical to choose the right control periods in order to minimize risk of bias, both in regards to duration and number (30). We therefore conducted a number of sensitivity analyses exploring this. By selecting control periods close in time to the case period and matching by week day (in case eg, Mondays had a higher level of sickness absence), we reduced some time-variant confounding similar to previous studies (32). To challenge our choice of 7 days between control periods, we tested 5 or 10 days between control periods. Results showed higher odds of sickness absence in all analyses. The risk estimate was highest when including 7 days between control periods, indicating effect of weekday. In the main analyses, we *a priori* selected case periods and control periods of 2 days as night shift work often is followed by a day off, where sickness absence is not recorded in the payroll data. To challenge this, we conducted analyses with case and control periods of 1 or 3 days. When restricting to 1 day, the results became statistically insignificant, when including 3 days, results were close to those of 2 days. Hence, there might be a risk of missing associations if choosing a case period that does not cover a period with full information.

When challenging the number of control periods, results showed lower risk estimates when choosing 1 period compared to the 5 periods chosen *a priori*. However, choosing more periods eg, 6 or 9, did not affect the results. This is supported by the literature, where previous studies have found stable estimates when including ≥5 control periods (36). We excluded participants with sickness absence 90 days prior to the case period in order to avoid bias due to previous sickness absence. However, sensitivity analyses showed no differences in risk estimates when not doing so.

### Strengths and limitations

The case-crossover design meets many of the limitations related to previously used study designs eg, vulnerability to between-subject differences. Further, by using DWHD, we were able to include a large number of participants directly from the database, yielding a 100% participation rate during follow-up. The database includes the accurate daily information on working time and sickness absence, both of which have been validated (2). We thereby excluded recall bias in relation to both working time and sickness absence.

Some limitations to the study need to be addressed. As in all observational studies addressing night and shift work, there is a risk of selection bias in terms of a survivor population, ie, a population of night workers who have the highest tolerance for night and shift work, and thereby a risk of affecting comparability between groups of night and non-night shift workers. There is therefore also a risk of selection bias. However, the advantages using the case-crossover design, where a person is her or his own control along with objective exposure assessment gives us the possibility to handle bias due to misclassification of exposure and therefore increase the exchangeability.

The study includes a very high percentage of women, which usually decreases the possibility of generalizing the results in regards to sex. However, due to the large population size, there is still almost 1200 cases among men, and the results from the stratified analyses showed higher odds of sickness absence for both men and women. Accordingly, we have no reasons to believe results would be very different among men.

The case-crossover design allowed us to handle time-invariant confounding such as sex and occupation along with, to some extent, personality, genetic differences etc. as we assume that these factors did not change during the 56 days of the control periods. Still, it may be the case that there are time-varying differences between the case and control periods that are not accounted for by the case-crossover design, eg, task and workload. This would add to residual confounding in regards to the biological mechanisms, where task and workload could be considered confounders. However, differences in tasks and workload could also be considered as part of the mechanism and then, consequently, should not be regarded as confounding. Further, the use of hospital employees only may limit generalization to other professions as differences in workload in day versus night might not be the same issue in other sectors.

### Concluding remarks

This large and unique study among Danish hospital employees indicates that the risk of calling in sick is affected by the type of shift worked prior to the sick listing, independently of sex, age and time-invariant confounders. Extensive testing showed that methodological choices were important to consider when choosing the case-crossover design.

### Ethical approval

Approval from the Danish National Committee on Biomedical Research Ethics is not required for Danish questionnaire- and register-based studies (33).

### Funding

The National Research Centre for the Working Environment, Denmark funded this study under the Governmental grant for research on sickness absence. Further, the study was based on data reprocessed in prior projects funded by NordForsk, Nordic Program on Health and Welfare [grant number 74809] and the Danish Working Environment Research Fund [grant number 23-2012-09/20120220951]. No one but the authors had a role in planning, executing and interpretation of the study or in the decision of publishing.

The authors declare no conflicts of interest.
